# Improvement of Robot Accuracy with an Optical Tracking System

**DOI:** 10.3390/s20216341

**Published:** 2020-11-06

**Authors:** Ying Liu, Yuwen Li, Zhenghao Zhuang, Tao Song

**Affiliations:** 1 Shanghai Key Laboratory of Intelligent Manufacturing and Robotics, School of Mechatronic Engineering and Automation, Shanghai University, Shanghai 201900, China; liuying58@i.shu.edu.cn (Y.L.); prette@shu.edu.cn (Z.Z.); 2 Shanghai Robot Industrial Technology Research Institute, Shanghai 200062, China

**Keywords:** robot, kinematic parameter identification, optical tracking system

## Abstract

Robot positioning accuracy plays an important role in industrial automation applications. In this paper, a method is proposed for the improvement of robot accuracy with an optical tracking system that integrates a least-square numerical algorithm for the identification of kinematic parameters. In the process of establishing the system kinematics model, the positioning errors of the tool and the robot base, and the errors of the Denavit-Hartenberg parameters are all considered. In addition, the linear dependence among the parameters is analyzed. Numerical simulation based on a 6-axis UR robot is performed to validate the effectiveness of the proposed method. Then, the method is implemented on the actual robot, and the experimental results show that the robots can reach desired poses with an accuracy of ±0.35 mm for position and ±0.07° for orientation. Benefitting from the optical tracking system, the proposed procedure can be easily automated to improve the robot accuracy for applications requiring high positioning accuracy such as riveting, drill, and precise assembly.

## 1. Introduction

Industrial robots have been widely applied for manufacturing automation in high-volume production due to their good task repeatability features. In a common scenario of the use of these machines, a human operator teaches the robot to move to a desired position; the robot records this position and then repeats the taught path to complete the task. However, robot teaching is usually time-consuming for low-volume applications. Although offline programming can significantly reduce the workload for robot teaching, the generated robot paths are based on the robot’s nominal kinematic model and, therefore, whether the robot can successfully complete the task via offline programming depends on its absolute accuracy. The robot manufacturers provide the nominal values of the Denavit-Hartenberg (D-H) parameters of the robot. However, the actual values of these parameters can deviate from their nominal values due to the errors in manufacturing, assembly, etc., which accordingly cause positioning errors to the robot end-effector. As a result, the absolute accuracy of industrial robots is relatively low compared with many other types of manufacturing equipment such as CNC machine tools [1]. As a result, industrial robots still face challenges in many low-volume applications where high absolute accuracy (with the positioning error less than 0.50 mm for an industrial robot of a medium to large size) is required, such as milling, drilling, and precise assembly.

Kinematic calibration is a significant way to improve the absolute accuracy of robots [2]. Two types of calibration methods are available based on measurement methods. One is the open-loop calibration in which the absolute position and orientation of the robot end-effector are measured; the other is closed-loop calibration in which the position and orientation of the end-effector are measured relative to another reference part or gauge.

For open-loop calibration, laser trackers have been adopted as the measurement device with different calibration algorithms. By using a laser tracker in [3,4,5,6,7], the absolute positioning accuracy of the robot can reach about 0.10–0.30 mm. Among them, the least squares technique is the most often applied one, which aims at minimizing the sum of squared residuals [4,5]. In [7], a new kinematic calibration method has been presented using the extended Kalman filter (EKF) and particle filter (PF) algorithm that can significantly improve the positioning accuracy of the robot. Thanks to the principle of data driven modeling, artificial neural network (ANN) has a promising application in modeling complex systems such as calibration [3,6]. Nguyen [3] combined a model-based identification method of the robot geometric errors and an artificial neural network to obtain an effective solution for the correction of robot parameters. In [6], a back propagation neural network (BPNN) and particle swarm optimization (PSO) algorithm have been employed for the kinematic parameter identification of industrial robots with an enhanced convergence response. Coordinate measuring machines (CMM) have also been used in open-loop robot calibration [8,9,10]. For example, Lightcap [10] has determined the geometric and flexibility parameters of robots to achieve significant reduction of systematic positioning errors. In [11], an optical CMM and a laser tracker have been combined to calibrate the ABB IRB 120 industrial robot, so that the mean and maximum position errors can be reduced from more than 3.00 mm and 5.00 mm to about 0.15 mm and 0.50 mm, respectively.

For closed-loop calibration, the calibration models are established by incorporating different types of kinematic constraints induced by the extra reference parts or gauges. By using gauges in [12,13,14], the absolute positioning accuracy of the robot also can reach about 0.10–0.30 mm. He [12] has used point constraints to improve the accuracy of si*x*-axis industrial robots. The robot parameters are calibrated by controlling a robot to reach the same location in different poses. In [13], a non-kinematic calibration method has been developed to improve the accuracy of a si*x*-axis serial robot, by using a linear optimization model based on the closed-loop calibration approach with multiple planar constraints. Joubair [14] has presented a kinematic calibration method using distance and sphere constraints effectively improving the positioning accuracy of the robot. In another category of closed-loop calibration methods, the kinematic constraints are induced by vision systems [15,16,17,18]. For example, Du [15] has developed a vision-based robot calibration method that only requires several reference images, and the mean positioning error was reduced from 5–7 mm to less than 2 mm. Zhang [18] has proposed a stereo vision based self-calibration procedure with the max position error and orientation errors reduced to less than 2.50 mm and 3.50°, respectively.

The calibration procedure is usually not specific to a certain task, it does not account for the influence of the robot end-effector and it is difficult to correct the errors due to structural elastic deformation and other factors. Other research works are focused on the online positioning error compensation, i.e.,, correcting the positioning error directly with the integration of an external metrology system. Online positioning error compensation is usually task-oriented and does not require a precise kinematics model [19,20,21,22,23,24,25,26,27,28,29]. For example, Jiang [20] has proposed an on-line iterative compensation method combining with a feed-forward compensation method to enhance the assembly accuracy of a robot system (MIRS) with the integration of a 6-DoF measurement system (T-Mac) to track the real-time robot movement. In [21], an online compensation method has been presented based on a laser tracker to increase robot accuracy without precise calibration. Yin [22] has developed a real-time dynamic thermal error compensation method for a robotic visual inspection system. The method is designed to be applied on the production line and correct the thermal error during the robotic system operation. In [23], an embedded real-time algorithm has been presented for the compensation of the lateral tool deviation for a robotic friction stir welding system. Shu [26] has presented a dynamic path tracking scheme that can realize automatic preplanned tasks and improve the tracking accuracy with eye-to-hand photogrammetry measurement feedback. A dynamic pose correction scheme has been proposed by Gharaaty [27] which adopts the PID controller and generates commands to the FANUC robot controller. In [28,29], the authors have adopted an iterative learning control (ILC) to improve the tracking performance of an industrial robot.

In summary, different offline calibration and online compensation methods to improve the absolute accuracy of robots have been reported in the literature. However, to achieve high precision (up to 0.30–0.50 mm), these methods have some drawbacks. For open-loop calibration, those methods usually require external metrology systems such as laser trackers and CMM that are costly and lack of flexibility for production automation. For closed-loop calibration, using gauges to calibrate requires manual teaching and is difficult to automate, which will affect the calibration efficiency; using vision systems to calibrate can be quite flexible for autonomous calibration, but the positioning error can be as high as a few millimeters limited by the precision of vision systems. For online compensation, most methods adopt a laser tracker as an external metrology system with a laser target mounted on the end-effector, which is costly and can be influenced by the visibility of the laser target. Moreover, most of the above methods do not take into account the influence of positioning accuracy caused by tools. For these reasons, improving absolute accuracy is still a bottleneck challenge for robotic applications in low-volume high-precision tasks.

To overcome the above issues, we propose an approach to correct the values of the kinematic parameters through the measurement of the actual end-effector poses via an optical tracking system. This approach integrates an optical tracking system and a rigid-body marker (with multiple marker targets) mounted on the end-effector for online compensation of robot positioning error. The optical tracking system enables online measurement of the 6-DOF motion of the robot end-effector. In the parameter identification process, the influence of the tool, the positioning error of the robot base and the error in the D-H parameters are all considered. A least-square numerical algorithm is then used to correct the errors in these kinematic parameters of the robot. Compared with laser trackers, the cost of an optical tracking system is significantly lower than that of a laser tracking system. Also, the optical tracking system can simultaneously measure the poses of multiple markers attached on the measured object, which brings good visibility of the object and flexibility for integration to the optical tracking system. Compared with closed-loop calibration with gauges, the optical tracking system is convenient and easy to automate to improve the calibration efficiency. Compared with online compensation with laser trackers, this method is more cost effective and can provide better visibility of the robot tools. In summary, the contribution of this work is to propose a comprehensive method to correct not only the errors in the D-H parameters but also the positioning errors in the base and tool frames for the kinematic model, which by adopting the optical tracking system, can lead to an efficient and automatic calibration of the robotic system for the robot users.

In the rest of the paper, the system setup as described in Section 2, the detailed theoretical development is presented in Section 3 and Section 4 for the robot kinematics and the calibration of kinematic parameters respectively and then simulation and experimental results are given in Section 5 and Section 6, respectively.

## 2. System Description

### 2.1. System Setup

**Figure 1:**
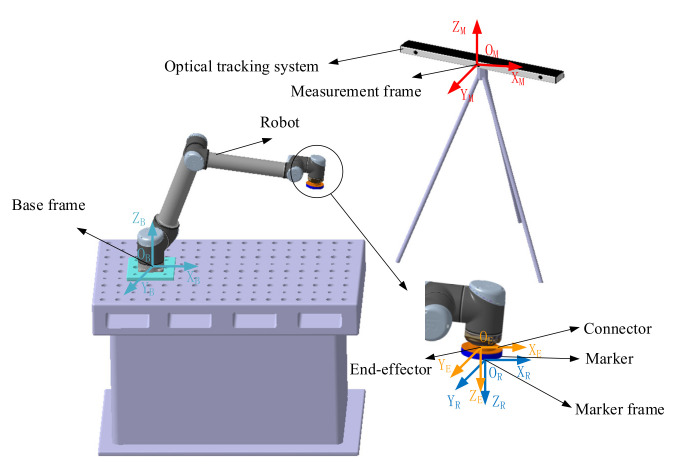
Robot calibration system configuration.

As illustrated in Figure 1, the system under study is composed of a serial robot, a tool mounted on the robot, and an optical tracking system. The robot can be programmed to drive the tool to move along a given path. The pose (position and orientation) of the tool can be obtained through the optical tracking system.

**Figure 2:**
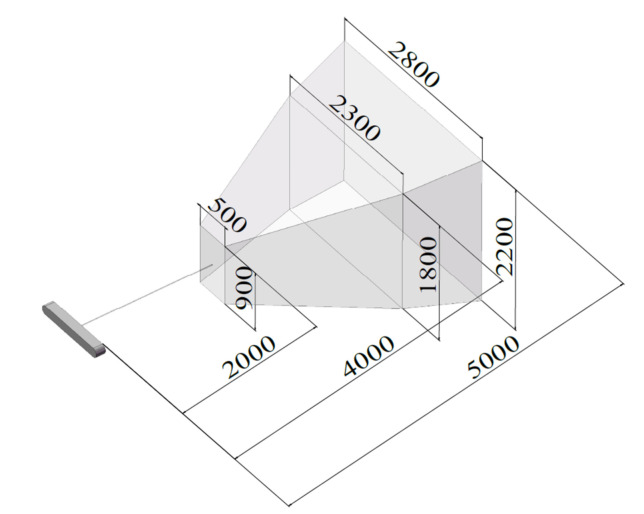
Measurement range of the optical tracking system used in this work (unit: mm).

More specifically, a Camsense S optical tracking system (Camsense, Shenzhen, China) is adopted in this system. It is a high-precision dynamic tracking device customized for precision robotic applications which can localize the 3-DOF positions of single-point markers and 6-DOF poses (position and orientation) of rigid-body markers moving in space in real time. The optical tracking system has been calibrated by measuring an object mounted on a CMM. The measuring range of this optical tracking system is 2 to 5 m as drawn in Figure 2. The positioning accuracies in the measuring ranges of 2 to 3 m, 3 to 4 m and 4 to 5 m are 0.08 mm, 0.14 mm and 0.21 mm, respectively. The optical tracking system uses two infrared CCD cameras working at a frame rate of 40 Hz. The camera and the markers are synchronized through 2.40 G wireless network, hence to enable the markers to work under the pulse mode and further to increase the signal noise ratio. The marker is wireless and contains multiple active light sources with infrared diodes emitting light at prescribed frequencies, thus they can be easily identifiable and not sensitive to external light conditions. A power source is integrated with the marker, and wireless communication can be established between the marker and the controller of the optical tracking system. Moreover, the markers are light and compact, for example, the rigid-body markers are with a mass of about 400 g, a diameter of 136 mm and a height of 16.5 mm.

Benefitting from the fact that the markers of the optical tracking system are light, compact and equipped with an internal power source, these markers can be easily attached on the robot end-effector during the measurement. By this means, the influence of the end-effector on the robot accuracy can be taken into account using this approach. Also, the optical tracking system can simultaneously measure the poses of multiple markers attached on the end-effector to guarantee the visibility of the end-effector by the tracking system.

Compared with laser tracking systems, the optical tracking system provides smaller measurement volume and they are slightly less accurate, but they are much less expensive and more flexible for automation. Also, the optical tracking can already provide sufficient accuracy for the machining and assembly tasks that are targeted in this work with a positioning accuracy requirement of about 0.30–0.50 mm. Moreover, since the repeatability of a medium to large-size industrial robot for the targeted tasks is about 0.10–0.20 mm, it would be difficult to improve the robot accuracy better than its repeatability by calibration even with a laser tracking system.

### 2.2. System Integration

**Figure 3:**
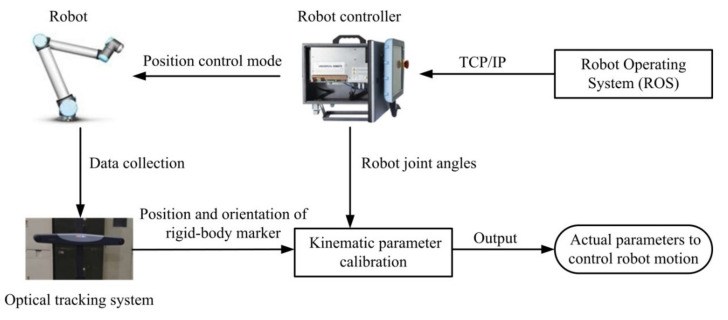
System integration diagram.

The integration of the optical tracking system with the UR robot used in this work is illustrated in Figure 3. With TCP/IP communication protocol, a computer running on robot operating system (ROS) can communicate with the robot controller and send commands to move the robot in a position control mode. When the robot moves to a series of calibration points, the position and orientation of the rigid-body marker mounted on the end-effector is measured by the optical tracking system for each point, and the robot joint angles are recorded simultaneously. Then, these data are used to calculate the actual kinematic parameters of the robotic system. Finally, these actual parameters can be output and used to calculate the robot kinematics for controlling the robot motion. The above procedure can be completed without human intervention, and thus provide the feasibility of autonomous calibration with the tool mounted on the robot.

## 3. Kinematic Modeling

### 3.1. Errors in the D-H Parameters

**Figure 4:**
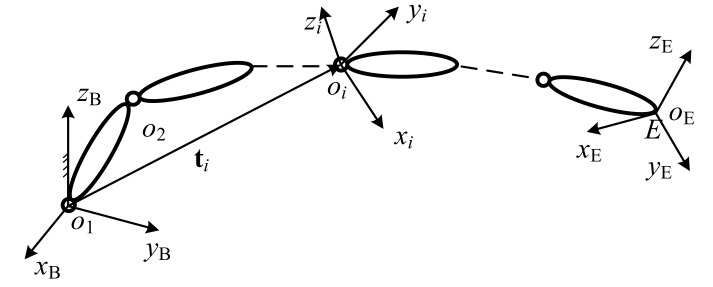
Kinematic scheme of a serial robot.

The kinematic scheme of a typical serial robot with *m* links and *m* revolute joints is plotted in Figure 4. The position vector of the *i*^th^ joint in the base frame {*O_X*_B_*Y*_B_*Z*_B_} is denoted by **t***_i_*. A local frame {*o_i__x_i_y_i_z_i_*} is established on each of the links with the origin at the *i*^th^ joint. The tip of the tool mounted on the last link is denoted as E, with its position vector written in the base frame as **t**_E_.

**Figure 5:**
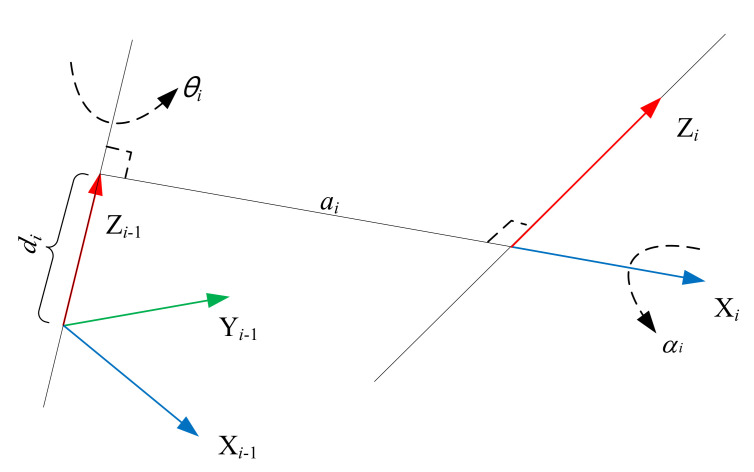
Definition of D-H parameters.

The homogeneous matrix of the *i*^th^ body frame {*o_i__x_i_y_i_z_i_*} relative to the (*i*-1)^th^ body frame can be given as follows:

(1) $${}_{i}^{i-1}T=\left[\begin{array}{cccc} \cos\theta_{i} & -\sin\theta_{i}\cos\alpha_{i} & \sin\theta_{i}\sin\alpha_{i} & a_{i}\cos\theta_{i}\\ \sin\theta_{i} & \cos\theta_{i}\cos\alpha_{i} & -\cos\theta_{i}\sin\alpha_{i} & -a_{i}\sin\theta_{i}\\ 0 & \sin\alpha_{i} & \cos\alpha_{i} & d_{i}\\ 0 & 0 & 0 & 1 \end{array}\right],$$

where *a_i_*, *d_i_*, *α_i_* and *θ_i_* are the D-H parameters of the *i*^th^ link as described in Figure 5, in which *a_i_* is the link length, *d_i_* is the link offset, *α_i_* is the link twist angle, and *θ_i_* is the joint angle. Then the homogeneous matrix of the end-effector {*O_X*_E_*Y*_E_*Z*_E_} relative to the robot base frame {*O_X*_B_*Y*_B_*Z*_B_} can be calculated as:

(2) $${}_{E}^{B}T_{n}=g(x_{m},p_{n}),$$

where the nonlinear function *g* represents robotic forward kinematics, subscript *n* denotes nominal value, and $x_{m}\in R^{m\times 1}$ is a vector of the joint variables calculated with nominal D-H parameters $p_{n}$, i.e.,:

(3) $$p_{n}={\left[\begin{array}{ccccccccc} \alpha_{1} & a_{1} & d_{1} & \theta_{1} & \cdots & \alpha_{m} & a_{m} & d_{m} & \theta_{m} \end{array}\right]}^{T}.$$

Vector $p_{n}$ contains the 4 *m* nominal D-H parameters, in which *m* is the number of the links. For a 6-axis industrial robot, vector $p_{n}\in R^{24\times 1}$ contains 24 parameters in total.

If we consider the errors in the D-H parameters, then the forward kinematics of the robot can be re-written as:

(4) $${}_{E}^{B}T=g(x_{m},p_{n}+\Delta p)\;\;,\;\;p_{a}=p_{n}+\Delta p.$$

Vector Δp contains the errors in the D-H parameters, and $p_{a}$ contains the actual D-H parameters. The errors of the D-H parameters can be caused by many factors. For example, the errors in the joint encoders can affect the values of the $\theta_{i}$ parameters in the D-H description.

### 3.2. Errors in the Marker Frame

In order to determine the pose relationship of the local frame fixed on the rigid-body marker relative to the end effector, the nominal pose of the marker frame with respect to the end-effector frame is written as:

(5) $${}_{R}^{E}T_{n}=\left[\begin{array}{cc} {}_{R}^{E}R_{n} & {}_{R}^{E}t_{n}\\ 0 & 1 \end{array}\right],$$

where ${}_{R}^{E}R_{n}$ and ${}_{R}^{E}t_{n}$ represent the nominal rotation matrix and nominal position vector between the end-effector tool frame and the rigid-body marker frame, respectively. In reality, the actual base frame ${}_{R}^{E}T_{n}$ can be inconsistent with the nominal base frame. We can use $\Delta {}_{E}^{R}T$ to represent the transformation relationship between the actual and normal frames, i.e.,:

(6) $${}_{R}^{E}T={}_{R}^{E}T_{n}\Delta {}_{R}^{E}T,$$

(7) $$\Delta {}_{R}^{E}T=rotx(\Delta \alpha_{\mathrm{ER}})roty(\Delta \beta_{\mathrm{ER}})rotz(\Delta \gamma_{\mathrm{ER}})trans(\Delta x_{\mathrm{ER}},\Delta y_{\mathrm{ER}},\Delta z_{\mathrm{ER}}),$$

where $\Delta \alpha_{\mathrm{RE}}$, $\Delta \beta_{\mathrm{RE}}$ and $\Delta \gamma_{\mathrm{RE}}$ represent the errors in the rotation angles of the marker frame with respect to the *x*, *y* and *z* axes of the end-effector frame, $\Delta x_{\mathrm{MB}}$, $\Delta y_{\mathrm{MB}}$ and $\Delta z_{\mathrm{MB}}$ represent the errors of the origin coordinates of the marker frame with respect to the end-effector frame.

### 3.3. Errors in the Base Frame

The nominal pose of the robot base frame with respect to the measurement frame cannot be obtained directly. However, the nominal D-H parameters of the robot are known, and the nominal pose of the marker frame with respect to the end-effector also is known. For a given robot configuration, the pose of the marker frame with respect to the measurement frame can be measured by the optical tracking system within the measurement range. Therefore, the nominal pose of the robot base frame with respect to the measurement frame can be obtained as follows:

(8) $${}_{B}^{M}T_{n}={}_{R}^{M}T{\left({}_{E}^{B}T_{n}{}_{R}^{E}T_{n}\right)}^{-1}.$$

Similarly, in order to determine the pose relationship of the actual robot base frame relative to the measurement frame, the nominal pose of the actual base frame with respect to the measurement frame is written as:

(9) $${}_{B}^{M}T={}_{B}^{M}T_{n}\Delta {}_{B}^{M}T,$$

(10) $$\Delta {}_{B}^{M}T=rotx(\Delta \alpha_{\mathrm{MB}})roty(\Delta \beta_{\mathrm{MB}})rotz(\Delta \gamma_{\mathrm{MB}})trans(\Delta x_{\mathrm{MB}},\Delta y_{\mathrm{MB}},\Delta z_{\mathrm{MB}}),$$

where $\Delta \alpha_{\mathrm{MB}}$, $\Delta \beta_{\mathrm{MB}}$ and $\Delta \gamma_{\mathrm{MB}}$ represent the errors in the rotation angles of the base frame with respect to the *x*, *y* and *z* axes of the measurement frame, $\Delta x_{\mathrm{MB}}$, $\Delta y_{\mathrm{MB}}$ and $\Delta z_{\mathrm{MB}}$ represent the errors in the origin coordinates of the base frame with respect to the measurement frame.

### 3.4. Errors of the System

**Figure 6:**
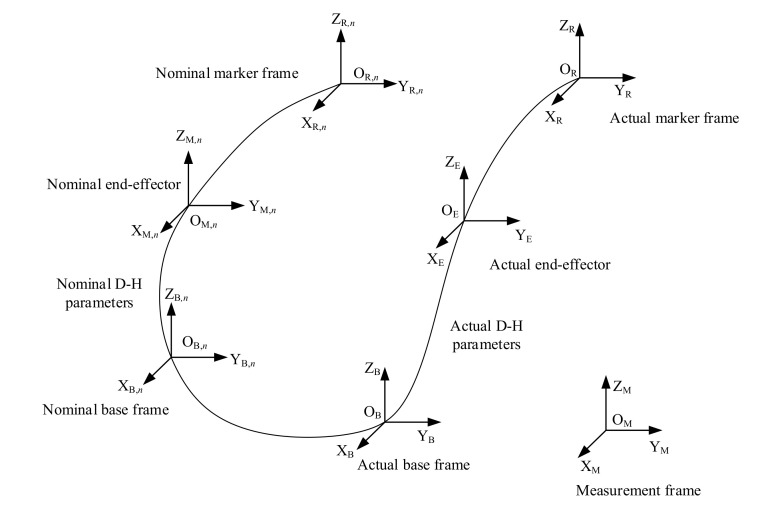
Nominal and actual coordinate frames.

In order to clearly show the systematic error, we establish coordinate frames for robot calibration as shown in Figure 6, where {$O_{M}X_{M}Y_{M}Z_{M}$} represents the measurement frame fixed on the laser tracking system, {$O_{B,n}X_{B,n}Y_{B,n}Z_{B,n}$} represents the nominal robot base frame, {$O_{B}X_{B}Y_{B}Z_{B}$} is the actual robot base frame, {$O_{E,n}X_{E,n}Y_{E,n}Z_{E,n}$} is the frame fixed on the end-effector obtained with the nominal D-H parameters, {$O_{E}X_{E}Y_{E}Z_{E}$} is the actual end-effector frame of the robot, {$O_{R,n}X_{R,n}Y_{R,n}Z_{R,n}$} represents the nominal frame fixed on the rigid-body marker, and {$O_{R}X_{R}Y_{R}Z_{R}$} represents the actual marker frame. According to the above relationship, introduce the error of each part into the system, we can get the following relationship:

(11) $${}_{R}^{M}T={}_{B}^{M}T_{n}\Delta {}_{B}^{M}T\;g(x_{m},p_{n}+\Delta p)\;{}_{R}^{E}T_{n}\Delta {}_{R}^{E}T.$$

Not only the errors in the D-H parameters but also the positioning errors of the robot base and the end-effector tool are considered in the calibration model in Equation (11).

## 4. Kinematic Parameter Identification

According to Equation (11), the pose of the marker frame relative to the measurement frame is a function of these kinematic parameters as:

(12) $$y_{\mathrm{MR}}=f(x_{\mathrm{MB}},y_{\mathrm{MB}},z_{\mathrm{MB}},\alpha_{\mathrm{MB}},\beta_{\mathrm{MB}},\gamma_{\mathrm{MB}},p_{n},x_{\mathrm{ER}},y_{\mathrm{ER}},z_{\mathrm{ER}},\alpha_{\mathrm{ER}},\beta_{\mathrm{ER}},\gamma_{\mathrm{ER}}),$$

where $y_{\mathrm{MR}}=[x_{\mathrm{MR}},y_{\mathrm{MR}},z_{\mathrm{MR}},\alpha_{\mathrm{MR}},\beta_{\mathrm{MR}},\gamma_{\mathrm{MR}}]$ represent position and orientation of the marker frame relative to the measurement frame. Taking the small disturbance for both sides of Equation (12), the model can be linearized as:

(13) $$\Delta y_{\mathrm{MR}}=J\Delta e,$$

(14) $$\Delta e={\left[\Delta x_{\mathrm{MB}},\Delta y_{\mathrm{MB}},\Delta z_{\mathrm{MB}},\Delta \alpha_{\mathrm{MB}},\Delta \beta_{\mathrm{MB}},\Delta \gamma_{\mathrm{MB}},\Delta p_{n}^{T},\Delta x_{\mathrm{ER}},\Delta y_{\mathrm{ER}},\Delta z_{\mathrm{ER}},\Delta \alpha_{\mathrm{ER}},\Delta \beta_{\mathrm{ER}},\Delta \gamma_{\mathrm{ER}}\right]}^{T},$$

where $\Delta y_{\mathrm{MR}}\in R^{6\times 1}$ represents the disturbance in the pose of the marker frame, $\Delta e\in R^{36\times 1}$ represents the disturbance in each parameter, and J is the 6 × 36 Jacobian matrix of the nonlinear kinematic model Equation (12) defined as follows:

(15) $$J=\left[\begin{array}{ccc} \frac{\partial f_{1}}{\partial x_{\mathrm{MB}}} & \ldots & \frac{\partial f_{1}}{\partial \gamma_{\mathrm{ER}}}\\ \vdots & \ddots & \vdots \\ \frac{\partial f_{6}}{\partial x_{\mathrm{MB}}} & \cdots & \frac{\partial f_{6}}{\partial \gamma_{\mathrm{ER}}} \end{array}\right].$$

This matrix can be numerically calculated by finite difference. For example, its element on the first row and the first column can be obtained as:

(16) $$J_{11}=\frac{\partial f_{1}}{\partial x_{\mathrm{MB}}}=\frac{\partial f_{1}(x_{\mathrm{MB}}+\Delta x_{\mathrm{MB}})-f_{1}(x_{\mathrm{MB}})}{\partial x_{\mathrm{MB}}}\;,$$

where $\Delta x_{\mathrm{MB}}$ is a small disturbance and we use a value of $1\times 10^{-6}$ in this work. We can compare the column vectors in the Jacobian matrix J. If two column vectors are identical, the errors in the two corresponding kinematic parameters have identical effect on the error in the end-effector pose.

According to Equation (11), matrix $\Delta {}_{B}^{M}T$ is generated from 6 variables as $\Delta y_{\mathrm{MB}}=\left[\Delta x_{\mathrm{MB}},\;\Delta y_{\mathrm{MB}},\;\Delta z_{\mathrm{MB}},\;\Delta \alpha_{\mathrm{MB}},\;\Delta \beta_{\mathrm{MB}},\;\Delta \gamma_{\mathrm{MB}}\right]$, $\Delta {}_{R}^{E}T$ is generated from 6 variables as $\Delta y_{\mathrm{ER}}=\left[\Delta x_{\mathrm{ER}},\;\Delta y_{\mathrm{ER}},\;\Delta z_{\mathrm{ER}},\;\Delta \alpha_{\mathrm{ER}},\;\Delta \beta_{\mathrm{ER}},\;\Delta \gamma_{\mathrm{ER}}\right]$, and vector Δp is generated from 4 *m* variables as the errors in the D-H parameter values. In this paper, multiple measurement data points are sampled for the pose of the marker frame relative to the measurement frame, and the robot joint angles are recorded simultaneously for each point. For each point, we have:

(17) $$y_{\mathrm{MR},k}=f(\Delta y_{\mathrm{MB}},\Delta p,\Delta y_{\mathrm{ER}},x_{m,k}),$$

where subscript *k* denotes the *k*^th^ sample point, *f* is the forward kinematics of the calibration system, and $x_{m,k}$ denotes the robot joint angles. In this study, a least-square numerical algorithm is applied to solve for the errors in the kinematic parameters, so the objective equation can be established as:

(18) $$(\Delta y_{\mathrm{MB}},\Delta p,\Delta y_{\mathrm{ER}})=\underset{\Delta y_{\mathrm{MB}},\Delta p,\Delta y_{\mathrm{ER}}}{\arg\;\min}∥f(\Delta y_{\mathrm{MB}},\Delta p,\Delta y_{\mathrm{ER}},x_{m,k})-y_{\mathrm{MR},k}∥,$$

where ∥ · ∥ represents the 2 norm. In the optimization process, we use the *fsolve* function with the Levenberg-Marquard algorithm for a numerical solution in MATLAB. Since the system kinematic parameters error is small, we set the initial values of all the kinematic parameter errors to be zero; the maximum number of iterations is set to be 500; the value of the function tolerance is set to be $1\times 10^{-6}$; and the value of the variable tolerance is set to be $1\times 10^{-6}$. The optimal solution is obtained as the system kinematic parameters error.

**Figure 7:**
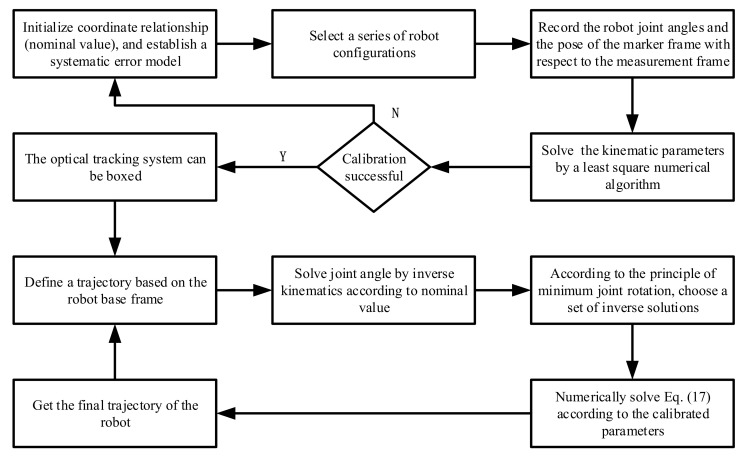
Specific process of the robot to accurately track the trajectory.

In summary, if we define a trajectory based on the robot base frame, the specific process of the robot to accurately track the trajectory is shown in Figure 7. After establishing the kinematics model, we can select a series of robot configurations to calibrate the robot. In order to implement our method on a robot, the direct and inverse kinematic problems should be solved for the robot on its controller using the corrected values of the kinematic parameters. By using the optical tracking system to measure the pose error, we can identify the kinematic parameters of the robot. According to the nominal value of the robot, we can solve the joint angle corresponding to the target trajectory. Substituting the joint angle obtained by the nominal value into the calibrated model, we can accurately reach the target position.

## 5. Simulation Study

### 5.1. Nominal Values

A simulation study is carried out to verify the above theoretical model. The simulated system is drawn in Figure 1. We define the nominal pose of the base frame relative to the measurement frame as:

(19) $${}_{B}^{M}T_{n}=\left[\begin{array}{llll} 1.00 & 0.00 & 0.00 & 2000.00(\mathrm{mm})\\ 0.00 & 1.00 & 0.00 & 2000.00(\mathrm{mm})\\ 0.00 & 0.00 & 1.00 & \;\;\;0.00\\ 0.00 & 0.00 & 0.00 & \;\;\;1.00 \end{array}\right]$$

The nominal pose of the marker frame relative to the end-effector is defined as:

(20) $${}_{R}^{E}T_{n}=\left[\begin{array}{l} 1.00\;0.00\;0.00\;\;\;\;\;0.00\\ 0.00\;1.00\;0.00\;\;\;\;\;0.00\\ 0.00\;0.00\;1.00\;100.00(\mathrm{mm})\\ 0.00\;0.00\;0.00\;\;\;\;\;1.00 \end{array}\right]$$

In this simulation, we use UR10 as an example. The nominal D-H parameters of the robot are shown as Table 1.

### 5.2. Giving Errors in Kinematic Parameters

In this simulation case, we define the errors in the base frame relative to the measurement frame in Table 2. We define the errors in the D-H parameters in Table 3. Also, we define the errors in the marker frame relative to the end-effector in Table 4. 

### 5.3. Simulation Result

In the calibration process, the position and orientation of the rigid-body marker is measured by the optical tracking system. In order to make the simulation closer to reality, we introduce random measurement errors into the measurement results. The position errors in the X, Y and Z directions are between −0.10 mm and +0.10 mm, the orientation errors in the X, Y and Z directions are between −0.10° and +0.10°. A total of 30 sets of data are selected with joint angles shown in Appendix A. Simultaneously, the actual poses of the marker frame with respect to the measurement frame can be obtained as shown in Appendix B. For each data point, we establish six equations; therefore, the errors in the required kinematic parameters can be obtained numerically. The pose errors of the base frame relative to the measurement frame are shown in Table 5. The errors in the D-H parameters are shown in Table 6. The pose errors of the marker frame relative to the end-effector are shown in Table 7. Through the above analysis, the parameter error after calibration can be compared with the parameter errors given in Table 2, Table 3 and Table 4. 

The comparison is shown in Table 8, with the given parameter error in the second column and the calculated parameter errors in the third column. It can be seen that the kinematic errors calculated from the theoretical model reasonably match the errors given in Section 5.2, which shows the effectiveness of the proposed method. A further investigation of the column vectors of the Jacobian matrix shows that some kinematic parameter errors cause identical effect on the end-effector pose. For instance, the third and the ninth column vectors in matrix J are identical, which represents that the kinematic parameter errors Δ$z_{\mathrm{MB}}$ and Δ*d*_1_ have identical effect on the positioning error of the effector, since the $z_{\mathrm{MB}}$ axis is parallel to the axis of the first joint. These are related to the geometric characteristics of the UR10 robotic system. For these parameters, their calculated errors after calibration do not guarantee to be close to their given errors, but the summation of their calculated errors is close to the summation of their given errors.

**Figure 8:**
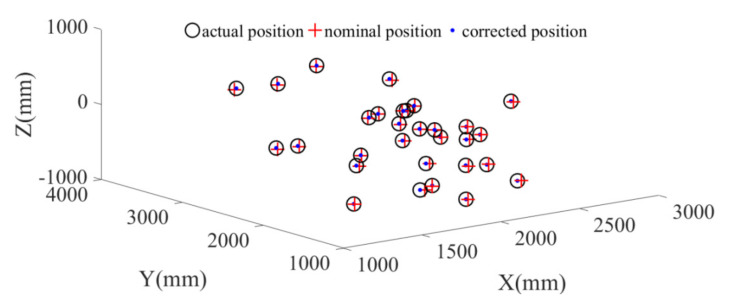
Nominal position and corrected positions.

**Figure 9:**
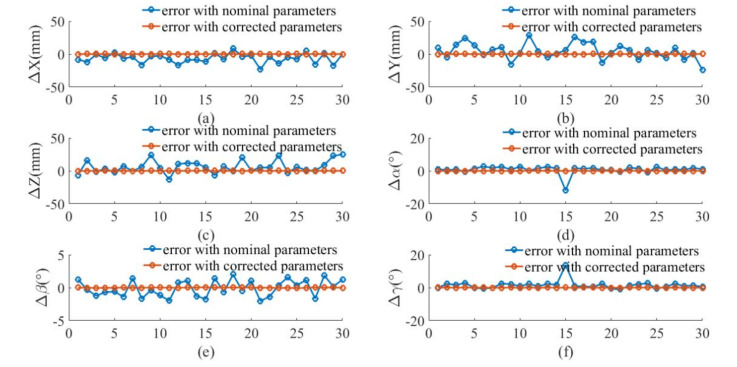
Pose errors before and after calibration for simulation: (**a**) X axis; (**b**) Y axis; (**c**) Z axis; (**d**) roll α; (**e**) pitch β; (**f**) yaw γ.

We proceed to demonstrate the improvement of the robot absolute accuracy in the simulation. According to the joint angles of the robot at the 30 data points, the positions of the marker frame relative to the measurement frame can be obtained from the forward kinematics with the nominal parameter values given in Section 5.1, which are called the nominal positions. After calibration, the positions of the marker frame relative to the measurement frame can be calculated by the corrected parameters, which are called the corrected positions. The actual positions of the marker frame relative to the measurement frame can be obtained from the forward kinematics with the actual parameter values given in Section 5.2, which are called the actual positions. The nominal positions and corrected positions are compared with the actual positions in Figure 8. It can be observed that the corrected positions are closer to the actual positions than the nominal positions. The differences between the nominal/corrected pose of the marker and the actual poses are displayed in Figure 9. It can be seen that the positioning accuracy of the robot is obviously improved after calibration.

The average error of marker poses at the 30 data points in each direction is used as the evaluation index, i.e.,:

(21) $$\Delta {\bar{y}}_{\mathrm{MR}}=\frac{\sum_{k=1}^{30}\left|y_{\mathrm{MR},k}-y_{\mathrm{MR},k,n}\right|}{30}.$$

Before calibration, the average error of the marker poses in each direction is shown in Table 9. After calibration, the average error of the marker poses in each direction is shown in Table 10. It is obvious that the error is significantly reduced in each direction.

We have also carried out a series of numerical simulation studies with different amount of errors in the D-H parameters. For the length variables, we set the error varying from −1 to +1 mm with a step of 0.05 mm; for the angle variable, we set the error varying from −1° to +1° with a step of 0.05°. For all the cases, the optimization process can converge and output the predefined error values.

## 6. Experimental Demonstration

### 6.1. Experimental Setup

An experimental study is given to demonstrate the validity of the proposed approach for a pin-hole insertion at multiple points. It is expected that after the robot is taught to insert the pin at limited points, it can complete the insertion automatically and smoothly at all the points. We will show that with nominal kinematic parameters, the robot is difficult to complete the task smoothly, while with corrected parameters, the insertion is performed much more smoothly.

**Figure 10:**
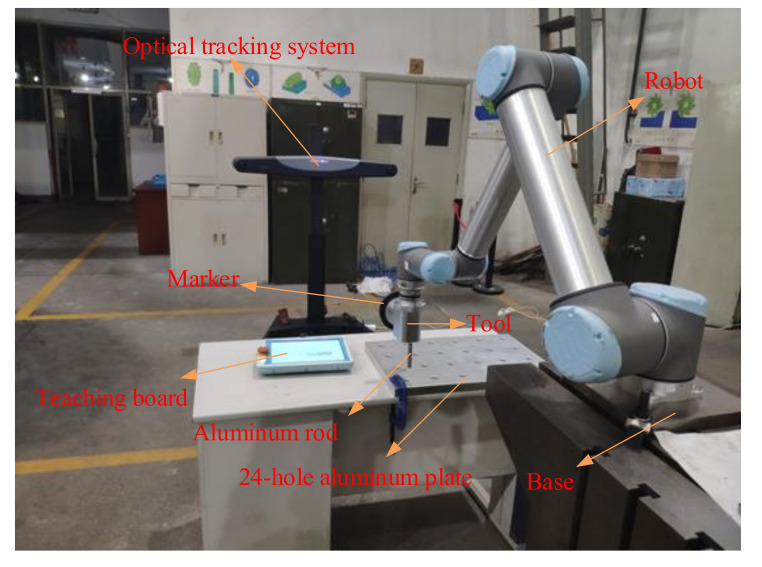
Experimental setup.

As shown in Figure 10, the experimental setup consists of a six-DOF UR10 robot fixed on a workbench, an optical tracking system fixed on the ground, a rigid marker, an adaptor plate, and a tool mounted on the robot. Also, an aluminum rod with a diameter of 20.00 mm and an aluminum plate with a length of 800.00 mm, a width of 600.00 mm and 24 holes are machined for the assembly task. The rod and the plate are shown in Figure 10.

### 6.2. Calibration Results

It is preferable to move the robot throughout the workspace and within the measurement range during the calibration process. For this optical tracking system, the measuring range is 2 to 5 m with a nominal volumetric positioning accuracy of 0.20 mm. As mentioned in Section 2, the measurement accuracy in the measurement range of 2 to 3 m is better than that in the range of 3 to 5 m, therefore the measurement distance of the experimental platform is within 2 to 3 m. We have carried out a series of other tests where the poses of the marker were measured at different data points. In these tests, the number of the data points varied from 30 to 40, and we found very similar results from these calibration tests. Using the above method, the pose of the base frame relative to the measurement frame are initially determined as:

(22) $${}_{B}^{M}T_{n}=\left[\begin{array}{l} -0.9996\;\;0.0123\;-0.0231\;\;\;\;\;\;-42.46(\mathrm{mm})\\ -0.0229\;\;0.0165\;\;\;\;\;\;0.9996\;\;\;\;-341.45(\mathrm{mm})\\ \;\;\;0.0127\;\;0.9998\;-0.0162\;\;-3739.49(\mathrm{mm})\\ \;\;\;0.00\;\;\;\;\;\;\;\;0.00\;\;\;\;\;\;\;\;\;\;\;0.00\;\;\;\;\;\;\;\;\;\;\;\;1.00 \end{array}\right]\;.$$

Also, the pose relationship between the marker frame and the end-effect can be initially determined from their CAD model, i.e.,:

(23) $${}_{R}^{E}T_{n}=\left[\begin{array}{l} 1.00\;\;\;\;\;\;0.00\;\;\;0.00\;\;\;\;\;\;0.00\\ 0.00\;\;\;\;\;\;0.00\;\;\;1.00-98.50(\mathrm{mm})\\ 0.00\;\;\;-1.00\;\;\;0.00\;103.50(\mathrm{mm})\\ 0.00\;\;\;\;\;\;0.00\;\;\;0.00\;\;\;\;\;\;1.00 \end{array}\right]\;.$$

During the calibration process, we recorded the joint angles of the robot at 30 data points as shown in Appendix C. The poses of the marker relative to the measurement frame are obtained by the optical tracking system and listed in Appendix D. By using the proposed calibration method, the pose error of the base frame relative to the measurement frame is calculated and shown in Table 11. The D-H parameter error obtained by the solution is shown in Table 12. The pose error of the marker frame relative to the end-effector frame is shown in Table 13.

**Figure 11:**
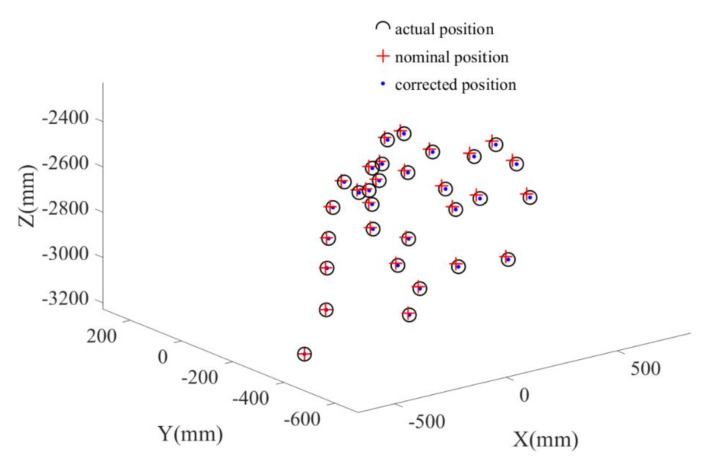
The nominal and corrected positions.

**Figure 12:**
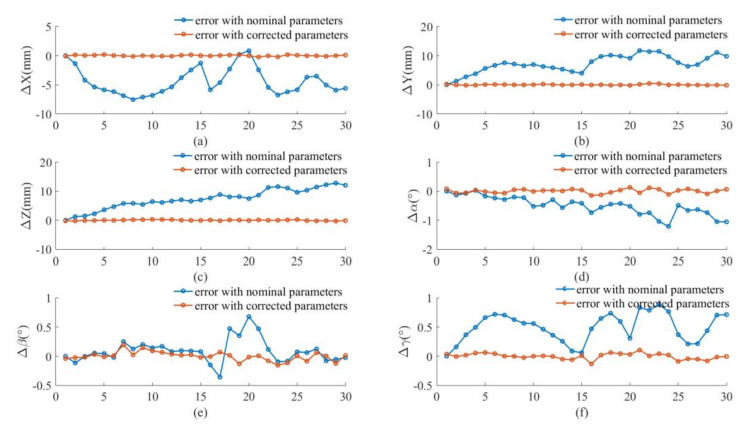
Pose errors before and after calibration for test: (**a**) X axis; (**b**) Y axis; (**c**) Z axis; (**d**) roll  α; (**e**) pitch  β; (**f**) yaw  γ.

The nominal positions (calculated from forward kinematics with nominal kinematic parameters) and corrected positions (calculated from forward kinematics with corrected kinematic parameters) are compared with actual positions (measured by optical tracking system) are compared Figure 11. It can be observed that the corrected positions are closer to the actual positions than the nominal positions. The pose errors before and after calibration are shown in Figure 12 for the calibration points. The average error at these points each direction before and after calibration is shown in Table 14 and Table 15 respectively. It is seen that the positioning accuracy of the robot can be dramatically improved for each direction at these calibration points. For example, the position error is reduced from 2.00–3.00 mm to less than 0.20 mm.

**Figure 13:**
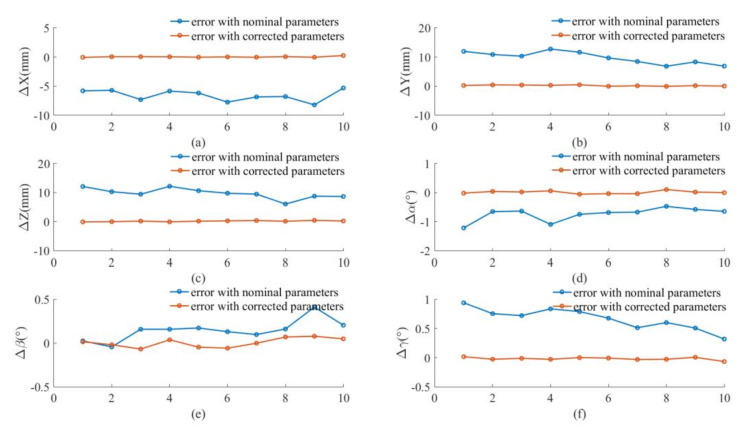
Pose errors before and after calibration of 10 data points: (**a**) X axis; (**b**) Y axis; (**c**) Z axis; (**d**) roll α; (**e**) pitch β; (**f**) yaw γ.

Furthermore, to verify the calibrated parameters, we randomly sampled 10 points rather than the calibration points and measured the poses of the marker at these random points. The joint angles for these points are shown in Appendix E and the measured pose of the marker relative to the measurement frame is shown in Appendix F. Substituting the joint angle of the robot into forward kinematics of the calibration system Equation (11) with nominal and corrected parameters, we can obtain the pose relationship of the marker frame relative to the measurement frame before and after calibration. The pose errors before and after calibration are compared in Figure 13 for these points. The average error of the marker pose in each direction before and after calibration is listed in Table 16 and Table 17, respectively. Again, we observe that the positioning accuracy of the robot can be dramatically improved for each direction at these randomly sampled points. It can be found that with the optical tracking system, the mean value of the position errors of the UR10 robot at the sampled points is improved to about 0.348 mm with a standard deviation of about 0.096 mm and the mean value of the angular errors is improved to about 0.070° with a standard deviation of about 0.024°. The resulting position accuracy is compared with the results obtained in some previous works on robot calibration in Table 18. It is observed is the position accuracy obtained using the proposed method is slightly lower than or similar to the accuracy after open-loop calibration with laser tracking system and CMM [4,7,11] and after closed-loop calibration with probes and gauges [13], and it is significantly higher than the accuracy after calibration with monocular and stereo vision cameras [15,18]. Note that the angular errors of the robots after calibration were not provided in these references. The robot accuracy improvement using the proposed method is limited by the measurement accuracy of the optical tracking system mentioned in Section 2.1, and it is also affected by the intrinsic characteristics of the robot such as the errors induced by non-kinematic factors like structural deformations. However, the proposed method can already provide sufficient accuracy for many tasks targeted by this work with an accuracy requirement of about 0.30–0.50 mm. Moreover, benefited from the compact size, light weight and good visibility of the markers of the optical tracking system, the proposed method provides possibility for correcting the kinematic model for a whole robotic system with a specific tool mounted on the robot withouts human intervention, which is convenient and easy to automate.

### 6.3. Insertion Results

**Figure 14:**
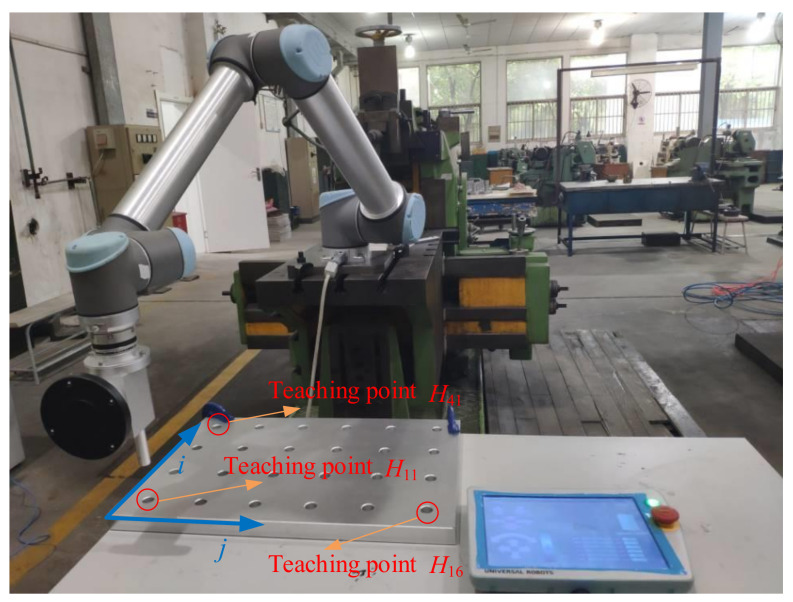
Teaching the robot insertion at three points.

We proceed to perform the insertion task with the robot. In the experiment, the robot is programmed to perform multiple pin-hole insertion tasks in a position control mode. If the robot experiences small positioning error, the pin can be well aligned with the holes and then successfully inserted into the hole. The diameter of the holes on the aluminum plate hole is inspected as 20.15 mm, and the rod diameter is inspected as 19.85 mm. To insert the aluminum rod into the holes, it is first necessary to determine the relationship between the base frame and the plate. For this purpose, three holes (not on the same line) are selected in the aluminum plate. Then the robot is taught to insert the pin into these holes using a traditional teaching method, and we can obtain the positional relationship of the teaching point relative to the base frame. As shown in Figure 14, in this experiment, we select points *H*_11_, *H*_41_ and *H*_16_ for teaching the robot. After teaching, we can obtain the positions of the **t**_11_, **t**_41_ and **t**_16_ relative to the robot base through the forward kinematics with recorded joint angles at these points. Then the position of the point on the *i*^th^ row and *j*^th^ column on the plate relative to the base can be obtained through three points, i.e.,:

(24) $$t_{ij}=t_{11}+(\frac{t_{16}-t_{11}}{5})(j-1)+(\frac{t_{41}-t_{11}}{3})(i-1).$$

When the robot reaches other positions, we maintain the same orientation for the end-effector as at the first teaching point *H*_11_. By performing inverse kinematics, the joint angles for the robot moving to each position can be obtained.

**Figure 15:**
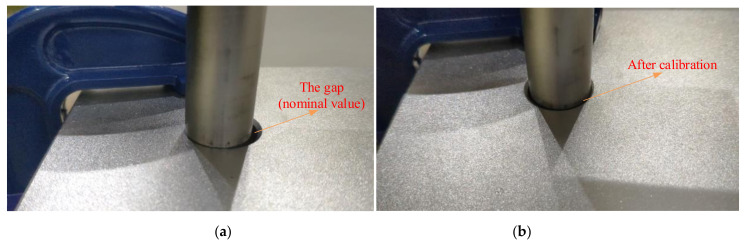
Alignment of rod and hole (**a**) before calibration and (**b**) after calibration.

**Figure 16:**
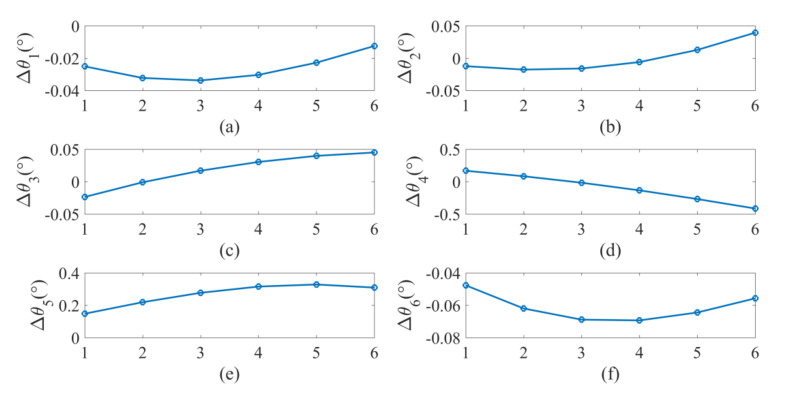
Difference between the joint angles before and after calibration: (**a**) joint 1; (**b**) joint 2; (**c**) in joint 3; (**d**) joint 4; (**e**) joint 5; (**f**) joint 6.

**Figure 17:**
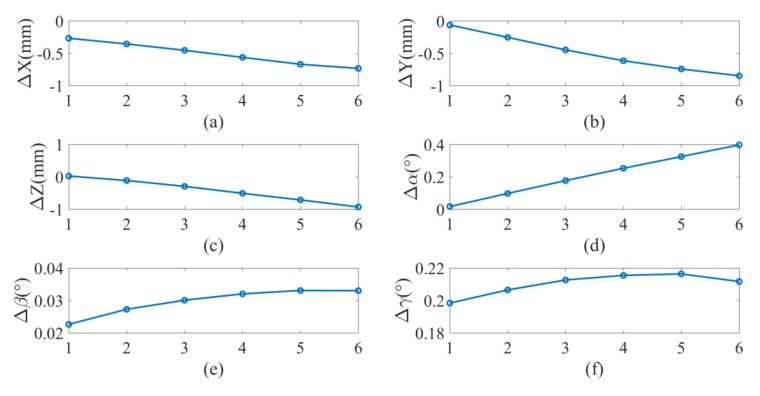
Difference between the end-effector position before and after calibration: (**a**) X axis; (**b**) Y axis; (**c**) Z axis; (**d**) roll α; (**e**) pitch β; (**f**) yaw γ.

**Figure 18:**
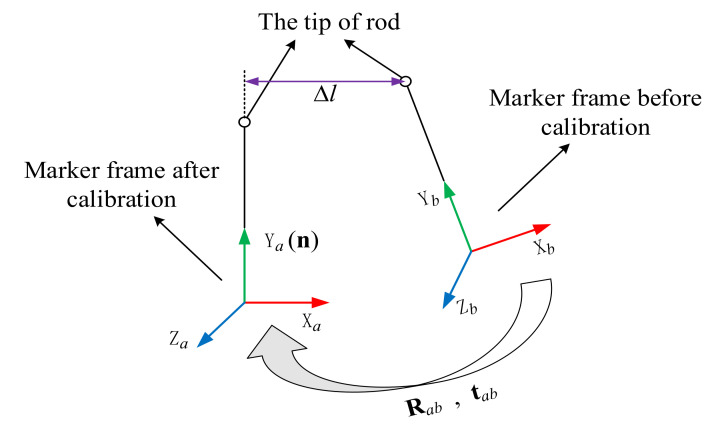
Misalignment of the tip.

**Figure 19:**
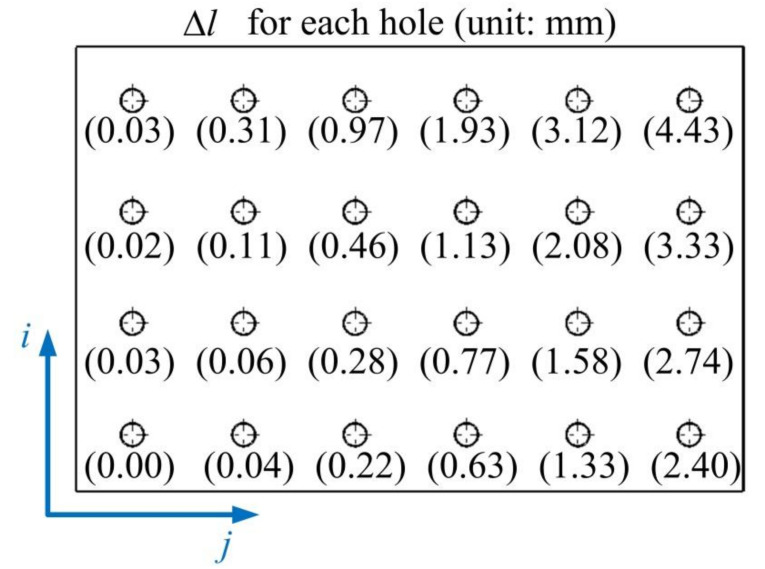
Corrected misalignment for each hole.

It is found that the pin can be smoothly inserted into each hole after calibration. However, with the nominal D-H parameters, the insertion is not smooth and can fail at some points. For example, the close observation at point *H*_44_ before and after calibration is shown in Figure 15. We can observe considerable misalignment between the tip of the pin and the centerline of the hole before calibration. To demonstrate the influence of positioning error correction on the actual robot motion, the joint angles of the robot before and after the calibration to reach the six holes on the third row of the workpiece are listed in Appendix G and Appendix H. The difference between the joint angles before and after calibration is shown in Figure 16. We can observe considerable joint angle difference when the corrected kinematic parameters are used to solve the inverse kinematics. Accordingly, the difference between the end-effector pose before and after calibration is displayed in Figure 17. Again, we observe considerable corrections in the *X* and *Y* directions and in the α angle which can significantly affect the smoothness of the insertion. We further analyze the corrected misalignment between the pin and the hole using the proposed method. As shown in Figure 18, we can define the orientation deviation as $R_{ab}$ and the position deviation as$\;t_{ab}$ for marker frame before and after calibration. The position displacements of the tip of the rod before and after calibration are denoted as:

(25) $$\Delta d=R_{ab}t_{tip}+t_{ab}-t_{tip},$$

where $t_{tip}$ is the position vector of the tip of the pin written in the marker frame. It is noted that the displacement along the direction of the hole does not affect the insertion task. Therefore, we can obtain the misalignment of the pin as follows:

(26) $$\Delta l=\left\|\Delta d-(\Delta d\;\cdot \;n)n\right\|,$$

where Δl is the misalignment, and n is the unit direction vector of along the direction of the hole. Figure 19 shows the misalignment corresponding to each hole on the part. In general, the larger the misalignment is, the more difficult it is to insert successfully. In this experiment, the points *H*_44_, *H*_45_ and *H*_46_ are not inserted successfully. A further observation shows the insertion forces acting on the robot in the vertical direction can be reduced from over 100 N before correction to less 2 N after correction for these holes.

## 7. Conclusions

In this work, we propose a method for the improvement of robot accuracy with an optical tracking system. Compared with existing methods using laser trackers, the proposed method has lower cost and is more flexible due to the advantages of the optical tracking system. In both the simulation and the experiment, the influence of the tool on the robot accuracy can be considered in this method, while most existing calibration methods are performed without the tool for the automation tasks. Furthermore, the proposed calibration procedure can be easily automated, in which the errors in the D-H parameters, the robot base position, and the tool position are all corrected. Instead of updating the kinematic parameters directly on the robot controller, the users can incorporate a separate controller to re-calculate the joint angles with the actual values of the kinematic parameters and then to send these joint angles to the robot to complete the motion. The proposed method provides possibility for a comprehensive calibration of a whole robotic system with a specific tool mounted on the robot. The procedure can be completed without human intervention and thus can be easily automated by robot users.

Simulation and experimental studies are performed to demonstrate the effectiveness of the proposed method for a UR10 robot. It is shown that the robot cannot complete an insertion task for multiple holes smoothly with nominal kinematic parameters by teaching a very limited number of points. However, using the proposed method, the robot can successfully complete the same task. This enables us to use the paths generated from offline programming to complete complicated tasks over a large work envelope. Although the simulation and experimental demo are done on a UR10 robot, it is expected that the proposed method can be extended to other industrial and collaborative serial robots. In future research, theoretical analysis on the convergence of the optimization process is necessary to evaluate the reliability of the final solution and using other equipment like a laser tracking system to calibrate the UR10 robot will be investigated for a comparison with the proposed method.

## Figures and Tables

**Table 1:** Nominal D-H parameters of the UR10 robot.

i	$\alpha_{i}\;({}^{\circ})$	$a_{i}\;(\mathrm{mm})$	$d_{i}\;(\mathrm{mm})$	$\theta_{i}\;({}^{\circ})$
1	0.00	0.00	127.30	0.00
2	90.00	0.00	0.00	0.00
3	0.00	−612.00	0.00	0.00
4	0.00	−572.30	163.90	0.00
5	90.00	0.00	115.70	0.00
6	−90.00	0.00	92.20	0.00

**Table 2:** Given errors in the base frame relative to the measurement frame.

$\Delta x_{\mathrm{MB}}\;(\mathrm{mm})$	$\Delta y_{\mathrm{MB}}\;(\mathrm{mm})$	$\Delta z_{\mathrm{MB}}\;(\mathrm{mm})$	$\Delta \alpha_{\mathrm{MB}}\;({}^{\circ})$	$\Delta \beta_{\mathrm{MB}}\;({}^{\circ})$	$\Delta \gamma_{\mathrm{MB}}\;({}^{\circ})$
0.50	0.50	0.50	0.50	0.50	0.50

**Table 3:** Given errors in the D-H parameters.

*i*	$\Delta \alpha_{i}\;({}^{\circ})$	$\Delta a_{i}\;(\mathrm{mm})$	$\Delta d_{i}\;(\mathrm{mm})$	$\Delta \theta_{i}({}^{\circ})$
1	0.30	0.30	0.30	0.30
2	0.30	0.30	0.30	0.30
3	0.30	0.30	0.30	0.30
4	0.30	0.30	0.30	0.30
5	0.30	0.30	0.30	0.30
6	0.30	0.30	0.30	0.30

**Table 4:** Given errors in the marker frame relative to the end-effect.

$\Delta x_{\mathrm{ER}}\;(\mathrm{mm})$	$\Delta y_{\mathrm{ER}}\;(\mathrm{mm})$	$\Delta z_{\mathrm{ER}}\;(\mathrm{mm})$	$\Delta \alpha_{\mathrm{ER}}\;({}^{\circ})$	$\Delta \beta_{\mathrm{ER}}\;({}^{\circ})$	$\Delta \gamma_{ER}\;({}^{\circ})$
0.50	0.50	0.50	0.50	0.50	0.50

**Table 5:** Pose error of the base frame relative to the measurement frame via calibration.

$\Delta x_{MB}\;(\mathrm{mm})$	$\Delta y_{MB}\;(\mathrm{mm})$	$\Delta z_{MB}\;(\mathrm{mm})$	$\Delta \alpha_{MB}\;({}^{\circ})$	$\Delta \beta_{MB}\;({}^{\circ})$	$\Delta \gamma_{MB}\;({}^{\circ})$
0.39	0.52	0.40	0.41	0.50	0.40

**Table 6:** Calculated D-H parameter errors via calibration.

*i*	$\Delta \alpha_{i}\;({}^{\circ})$	$\Delta a_{i}\;(\mathrm{mm})$	$\Delta d_{i}\;(\mathrm{mm})$	$\Delta \theta_{i}\;({}^{\circ})$
1	0.39	0.39	0.40	0.40
2	0.30	0.27	0.31	0.30
3	0.30	0.29	0.31	0.30
4	0.30	0.28	0.31	0.30
5	0.30	0.30	0.21	0.31
6	0.33	0.32	0.41	0.43

**Table 7:** Pose error of the marker frame relative to the end-effect via calibration.

$\Delta x_{ER}\;(\mathrm{mm})$	$\Delta y_{ER}\;(\mathrm{mm})$	$\Delta z_{ER}\;(\mathrm{mm})$	$\Delta \alpha_{ER}\;({}^{\circ})$	$\Delta \beta_{ER}\;({}^{\circ})$	$\Delta \gamma_{ER}\;({}^{\circ})$
0.55	0.57	0.42	0.57	0.56	0.43

**Table 8:** Given and calculated errors in the kinematic parameters.

Parameters	Given Parameter Error	Calculated Parameter Error
$\Delta x_{\mathrm{MB}}+\Delta a_{1}\;(\mathrm{mm})$	0.80	0.78
$\Delta y_{\mathrm{MB}}\;(\mathrm{mm})$	0.50	0.52
$\Delta z_{\mathrm{MB}}+\Delta d_{1}\;(\mathrm{mm})$	0.80	0.80
$\Delta \alpha_{\mathrm{MB}}+\Delta \alpha_{1}\;({}^{\circ})$	0.80	0.80
$\Delta \beta_{\mathrm{MB}}\;({}^{\circ})$	0.50	0.50
$\Delta \gamma_{\mathrm{MB}}+\Delta \theta_{1}\;({}^{\circ})$	0.80	0.80
$\Delta \alpha_{2}\;({}^{\circ})$	0.30	0.30
$\Delta \alpha_{3}\;({}^{\circ})$	0.30	0.30
$\Delta \alpha_{4}\;({}^{\circ})$	0.30	0.30
$\Delta \alpha_{5}\;({}^{\circ})$	0.30	0.30
$\Delta \alpha_{6}\;({}^{\circ})$	0.30	0.33
$\Delta a_{2}\;(\mathrm{mm})$	0.30	0.27
$\Delta a_{3}\;(\mathrm{mm})$	0.30	0.29
$\Delta \alpha_{4}\;({}^{\circ})$	0.30	0.28
$\Delta a_{5}\;(\mathrm{mm})$	0.30	0.30
$\Delta a_{6}\;(\mathrm{mm})$	0.30	0.32
$\Delta d_{2}+\Delta d_{3}+\Delta d_{4}\;(\mathrm{mm})$	0.90	0.93
$\Delta d_{5}\;(\mathrm{mm})$	0.30	0.21
$\Delta d_{6}+\Delta z_{\mathrm{ER}}\;(\mathrm{mm})$	0.80	0.84
$\Delta \theta_{2}\;({}^{\circ})$	0.30	0.30
$\Delta \theta_{3}\;({}^{\circ})$	0.30	0.30
$\Delta \theta_{4}\;({}^{\circ})$	0.30	0.30
$\Delta \theta_{5}\;({}^{\circ})$	0.30	0.31
$\Delta \theta_{6}+\Delta \gamma_{\mathrm{ER}}\;({}^{\circ})$	0.80	0.86
$\Delta x_{\mathrm{ER}}\;(\mathrm{mm})$	0.50	0.55
$\Delta y_{\mathrm{ER}}\;(\mathrm{mm})$	0.50	0.57
$\Delta \alpha_{\mathrm{ER}}\;({}^{\circ})$	0.50	0.57
$\Delta \beta_{\mathrm{ER}}\;({}^{\circ})$	0.50	0.56

**Table 9:** Average error of 30 data points before calibration.

$\Delta {\bar{x}}_{MR}\;(\mathrm{mm})$	$\Delta {\bar{y}}_{MR}\;(\mathrm{mm})$	$\Delta {\bar{z}}_{MR}\;(\mathrm{mm})$	$\Delta {\bar{\alpha }}_{MR}\;({}^{\circ})$	$\Delta {\bar{\beta }}_{MR}\;({}^{\circ})$	$\Delta {\bar{\gamma }}_{MR}\;({}^{\circ})$
7.82	10.23	8.59	1.64	1.14	1.74

**Table 10:** Average error of 30 data points after calibration.

$\Delta {\bar{x}}_{MR}\;(\mathrm{mm})$	$\Delta {\bar{y}}_{MR}\;(\mathrm{mm})$	$\Delta {\bar{z}}_{MR}\;(\mathrm{mm})$	$\Delta {\bar{\alpha }}_{MR}\;({}^{\circ})$	$\Delta {\bar{\beta }}_{MR}\;({}^{\circ})$	$\Delta {\bar{\gamma }}_{MR}\;({}^{\circ})$
0.02	0.02	0.02	0.03	0.03	0.02

**Table 11:** Pose error of the base frame relative to the measurement frame via calibration.

$\Delta x_{MR}\;(\mathrm{mm})$	$\Delta y_{MR}\;(\mathrm{mm})$	$\Delta z_{MR}\;(\mathrm{mm})$	$\Delta \alpha_{MR}\;({}^{\circ})$	$\Delta \beta_{MR}\;({}^{\circ})$	$\Delta \gamma_{MR}\;({}^{\circ})$
2.52	−8.17	−1.06	−0.25	−0.28	0.07

**Table 12:** D-H parameter errors via calibration.

*i*	$\Delta \alpha_{i}\;({}^{\circ})$	$\Delta a_{i}\;(\mathrm{mm})$	$\Delta d_{i}\;(\mathrm{mm})$	$\Delta \theta_{i}\;({}^{\circ})$
1	−0.27	2.49	−1.15	0.22
2	−0.02	0.34	−0.10	0.02
3	−0.51	−0.36	−0.34	0.01
4	−0.64	0.80	−0.19	0.14
5	−0.13	−0.64	−0.53	−0.04
6	0.15	−0.04	−0.59	0.60

**Table 13:** Pose error of the marker frame relative to the end-effect via calibration.

$\Delta x_{ER}\;(\mathrm{mm})$	$\Delta y_{ER}\;(\mathrm{mm})$	$\Delta z_{ER}\;(\mathrm{mm})$	$\Delta \alpha_{ER}\;({}^{\circ})$	$\Delta \beta_{ER}\;({}^{\circ})$	$\Delta \gamma_{ER}\;({}^{\circ})$
0.58	0.68	−0.37	−0.06	0.28	0.02

**Table 14:** Average error of 30 data points before calibration.

$\Delta {\bar{x}}_{MR}\;(\mathrm{mm})$	$\Delta {\bar{y}}_{MR}\;(\mathrm{mm})$	$\Delta {\bar{z}}_{MR}\;(\mathrm{mm})$	$\Delta {\bar{\alpha }}_{MR}\;({}^{\circ})$	$\Delta {\bar{\beta }}_{MR}\;({}^{\circ})$	$\Delta {\bar{\gamma }}_{MR}\;({}^{\circ})$
2.20	3.24	3.24	0.50	0.16	0.49

**Table 15:** Average error of 30 data points after calibration.

$\Delta {\bar{x}}_{MR}\;(\mathrm{mm})$	$\Delta {\bar{y}}_{MR}\;(\mathrm{mm})$	$\Delta {\bar{z}}_{MR}\;(\mathrm{mm})$	$\Delta {\bar{\alpha }}_{MR}\;({}^{\circ})$	$\Delta {\bar{\beta }}_{MR}\;({}^{\circ})$	$\Delta {\bar{\gamma }}_{MR}\;({}^{\circ})$
0.08	0.12	0.13	0.06	0.05	0.04

**Table 16:** Average error of 10 data points before calibration.

$\Delta {\bar{x}}_{MR}\;(\mathrm{mm})$	$\Delta {\bar{y}}_{MR}\;(\mathrm{mm})$	$\Delta {\bar{z}}_{MR}\;(\mathrm{mm})$	$\Delta {\bar{\alpha }}_{MR}\;({}^{\circ})$	$\Delta {\bar{\beta }}_{MR}\;({}^{\circ})$	$\Delta {\bar{\gamma }}_{MR}\;({}^{\circ})$
6.60	9.73	9.71	0.74	0.16	0.67

**Table 17:** Average error of 10 data points after calibration.

$\Delta {\bar{x}}_{MR}\;(\mathrm{mm})$	$\Delta {\bar{y}}_{MR}\;(\mathrm{mm})$	$\Delta {\bar{z}}_{MR}\;(\mathrm{mm})$	$\Delta {\bar{\alpha }}_{MR}\;({}^{\circ})$	$\Delta {\bar{\beta }}_{MR}\;({}^{\circ})$	$\Delta {\bar{\gamma }}_{MR}\;({}^{\circ})$
0.06	0.20	0.19	0.04	0.04	0.02

**Table 18:** Positioning error result comparison with previous works.

Reference	Methodology	Equipment	Robot	Mean (mm)	Std (mm)	Max (mm)
[4]	open-loop	laser tracking system	ABB IRB1600	0.364	0.130	0.696
[7]	open-loop	laser tracking system	Kawasaki RS10N	0.262	0.143	0.642
[11]	open-loop	laser tracker and CMM	ABB IRB120	0.146	0.065	0.437
[13]	closed-loop	probe and gauge	FANUC LR Mate 200iC	0.153	0.07	0.274
[15]	closed-loop	monocular vision	GOOGOL GRB3016	1.340	N/A	N/A
[18]	closed-loop	stereo vision	UR5	2.500	N/A	N/A
This work	open-loop	optical tracking system	UR10	0.348	0.096	0.467

## Appendix A

**Table A1:** Robot joint angles corresponding to 30 data points in the simulation.

	*θ*_1_ (°)	*θ*_2_ (°)	*θ*_3_ (°)	*θ*_4_ (°)	*θ*_5_ (°)	*θ*_6_ (°)
1	149.60	28.17	82.32	111.26	167.79	150.32
2	161.18	104.85	104.89	153.89	6.28	159.38
3	73.39	6.55	134.31	27.87	25.90	109.07
4	45.81	58.35	72.32	73.15	69.51	109.76
5	30.04	33.86	17.03	58.17	138.53	42.14
6	133.27	124.71	148.33	149.04	52.81	55.69
7	94.15	58.55	149.73	145.85	100.26	47.33
8	122.50	42.06	82.16	69.22	96.95	178.51
9	135.94	176.48	42.26	95.14	9.26	136.24
10	108.36	154.29	177.89	167.31	73.71	0.06
11	97.36	37.39	39.47	58.65	17.27	134.56
12	134.73	97.79	60.86	149.82	99.46	172.36
13	160.71	64.17	98.35	62.40	112.10	143.39
14	134.26	22.60	148.03	4.53	74.60	131.65
15	140.65	66.11	134.08	160.61	43.67	23.33
16	40.51	63.00	51.68	166.95	9.24	106.68
17	29.32	150.91	30.16	90.40	179.88	63.97
18	8.47	38.46	71.61	60.06	41.33	168.50
19	122.97	173.18	78.84	169.26	1.05	109.86
20	144.19	41.94	167.84	137.39	148.76	103.22
21	142.66	59.23	40.22	56.23	105.21	149.38
22	52.28	72.46	155.17	110.65	178.41	36.67
23	148.90	121.66	44.81	85.64	71.83	107.90
24	144.09	18.91	147.86	151.40	63.81	77.41
25	103.00	126.15	133.64	136.42	70.04	77.27
26	172.14	103.13	152.95	49.74	112.02	105.91
27	173.42	15.46	90.09	93.89	16.23	162.84
28	159.19	79.02	140.71	26.72	111.57	46.91
29	80.22	151.92	35.32	54.69	86.99	60.81
30	143.73	177.75	28.63	42.64	126.40	67.58

## Appendix B

**Table A2:** Actual poses of the marker relative to the measurement frame in the simulation.

	$x_{MR}\;(\mathrm{mm})$	$y_{MR}\;(\mathrm{mm})$	$z_{MR}\;(\mathrm{mm})$	$\alpha_{MR}\;({}^{\circ})$	$\beta_{MR}\;({}^{\circ})$	$\gamma_{MR}\;({}^{\circ})$
1	2313.22	1792.92	−589.74	80.57	−37.86	−77.55
2	1497.11	2537.32	−281.22	−89.89	24.78	−14.51
3	2304.66	1860.18	−206.67	−118.39	78.74	129.42
4	2277.65	1984.56	−645.25	−44.79	57.39	80.46
5	1390.68	1636.51	−742.07	−128.19	−11.44	98.47
6	1958.15	2445.07	12.88	−165.94	42.65	−2.39
7	2125.99	1999.70	−220.18	86.06	−4.69	47.70
8	2088.60	2107.16	−591.93	−70.43	−40.07	−169.87
9	1558.05	2900.97	414.29	−78.69	49.63	−95.51
10	2121.44	2318.46	101.56	−126.13	0.87	−10.79
11	2399.72	1582.72	−771.25	−121.84	64.98	123.51
12	1790.61	2386.28	−600.30	37.03	19.17	−151.35
13	1716.83	2187.95	−375.82	14.30	−47.02	−129.10
14	2010.64	2288.81	−89.58	−93.86	−29.99	129.54
15	2058.98	2339.72	−347.13	−106.54	81.21	−137.63
16	2109.96	1653.80	−936.88	79.96	39.42	36.70
17	2842.36	2528.24	−155.19	−88.56	−30.02	−26.84
18	1916.72	1693.40	−698.29	101.65	50.94	−26.05
19	1813.53	2922.75	560.58	−91.35	57.05	−5.05
20	2064.34	1952.87	−87.92	83.72	−4.21	113.01
21	2047.82	2100.22	−927.90	−141.50	−61.20	108.64
22	2067.28	2144.44	−134.59	−87.03	−55.04	−119.65
23	1396.18	2608.17	−294.57	−23.45	−4.68	164.83
24	2326.61	2073.32	−175.35	2.66	54.81	144.27
25	2129.31	2428.49	1.43	132.37	30.59	67.68
26	1939.25	2095.88	164.03	31.15	30.34	173.43
27	2446.71	2306.63	−458.95	−83.78	−10.46	−175.26
28	1765.21	2180.81	110.93	14.76	−26.74	125.14
29	2340.17	3051.32	158.13	−25.52	7.65	53.46
30	1165.36	2649.96	568.58	23.49	−34.46	136.14

## Appendix C

**Table A3:** Robot joint angles corresponding to 30 data points in the experiment.

	$\theta_{1}\;({}^{\circ})\;$	$\theta_{2}\;({}^{\circ})$	$\theta_{3}\;({}^{\circ})$	$\theta_{4}\;({}^{\circ})$	$\theta_{5}\;({}^{\circ})$	$\theta_{6}\;({}^{\circ})$
1	−152.81	−63.54	119.34	−143.66	−92.14	−61.12
2	−140.55	−47.11	103.77	−150.59	−85.43	−52.33
3	−130.90	−37.98	80.19	−131.04	−93.29	−41.23
4	−125.18	−37.92	75.17	−131.02	−92.88	−41.14
5	−120.02	−26.06	51.65	−117.04	−93.58	−21.81
6	−115.18	−24.36	47.40	−121.50	−90.64	−21.81
7	−109.32	−24.43	44.68	−113.53	−90.09	−21.82
8	−107.71	−35.02	62.02	−117.97	−90.30	−21.82
9	−109.97	−40.75	71.78	−124.13	−85.94	−21.82
10	−106.69	−49.33	86.08	−132.94	−89.67	−0.80
11	−108.77	−56.81	98.07	−132.92	−88.43	−0.94
12	−105.77	−55.93	104.87	−141.30	−92.75	−27.15
13	−107.46	−66.63	118.32	−141.23	−84.04	5.00
14	−109.04	−69.60	127.59	−141.44	−87.38	−25.52
15	−108.97	−81.65	139.60	−142.12	−95.35	−10.18
16	−99.83	−45.76	82.54	−143.31	−64.87	−10.33
17	−96.35	−24.46	59.62	−156.43	−92.18	−10.28
18	−96.30	6.48	−39.73	−53.90	−93.53	−10.27
19	−95.71	15.68	−68.46	−37.28	−88.28	−10.32
20	−94.43	10.49	−79.26	−11.56	−84.87	14.51
21	−88.27	4.61	−39.89	−53.28	−96.42	14.04
22	−88.09	−11.72	23.95	−96.94	−79.45	14.04
23	−84.94	−28.57	53.20	−119.15	−89.09	33.74
24	−85.48	−47.26	83.25	−135.46	−90.53	39.54
25	−89.56	−55.16	96.63	−133.57	−83.44	−14.82
26	−85.84	−68.78	118.18	−131.97	−85.53	20.92
27	−76.49	−60.10	114.03	−140.40	−90.71	−5.63
28	−77.15	−40.11	84.01	−141.30	−86.02	−5.62
29	−78.19	−28.77	56.12	−119.94	−86.48	30.33
30	−82.20	−41.33	82.48	−137.64	−93.58	43.06

## Appendix D

**Table A4:** Pose of the marker relative to the measurement frame in the experiment.

	$x_{MR}\;(\mathrm{mm})$	$y_{MR}\;(\mathrm{mm})$	$z_{MR}\;(\mathrm{mm})$	$\alpha_{MR}\;({}^{\circ})$	$\beta_{MR}\;({}^{\circ})$	$\gamma_{MR}\;({}^{\circ})$
1	−507.55	−343.78	−3164.85	−178.45	0.79	177.85
2	−491.67	−415.23	−2940.29	172.68	−2.47	178.62
3	−490.11	−417.03	−2755.51	−178.28	−1.16	−179.88
4	−416.83	−359.45	−2667.84	177.49	−6.72	−177.23
5	−412.02	−372.38	−2526.80	178.95	7.42	−178.07
6	−316.45	−333.96	−2453.84	171.29	2.55	−177.91
7	−179.45	−324.76	−2429.54	175.82	−3.49	179.15
8	−116.95	−297.38	−2511.32	177.93	−4.99	178.67
9	−135.61	−273.98	−2561.53	174.39	−2.73	175.35
10	−92.98	−248.51	−2644.52	173.07	15.00	179.56
11	−96.20	−255.28	−2749.69	177.73	16.91	177.18
12	−1.96	−312.83	−2788.46	177.78	−12.18	−178.31
13	−20.34	−286.53	−2913.43	−179.25	21.53	172.09
14	29.23	−329.69	−3007.87	−176.35	−7.50	174.06
15	14.00	−301.70	−3133.29	−174.04	7.71	−178.03
16	124.89	−199.61	−2577.55	158.68	−0.28	156.08
17	91.92	−325.38	−2421.07	148.59	−4.48	−176.66
18	84.14	−155.01	−2444.09	−177.59	−5.29	−178.76
19	123.90	−99.59	−2585.09	178.55	−5.73	176.42
20	126.93	−6.18	−2754.79	−169.25	17.10	172.35
21	213.06	−107.96	−2468.28	179.51	10.97	−175.52
22	287.68	−401.17	−2400.00	−172.56	10.84	167.44
23	288.67	−317.20	−2491.16	176.64	28.07	176.14
24	245.85	−241.94	−2659.00	171.44	34.59	177.31
25	277.83	−254.16	−2751.42	174.90	−15.93	171.45
26	253.96	−285.60	−2983.85	−171.48	15.71	174.52
27	418.63	−335.72	−2967.80	−177.40	−20.09	−179.90
28	491.96	−357.18	−2701.12	170.71	−18.84	172.30
29	444.17	−346.15	−2547.34	179.59	17.74	173.77
30	294.38	−334.62	−2669.74	171.82	34.70	−179.11

## Appendix E

**Table A5:** Robot joint angles for 10 random data points in the experiment.

	$\theta_{1}\;({}^{\circ})$	$\theta_{2}\;({}^{\circ})$	$\theta_{3}\;({}^{\circ})$	$\theta_{4}\;({}^{\circ})$	$\theta_{5}\;({}^{\circ})$	$\theta_{6}\;({}^{\circ})$
1	−84.55	−19.26	41.22	−116.82	−88.81	36.94
2	−91.87	−17.68	39.51	−116.13	−84.60	4.94
3	−93.04	−25.77	41.83	−101.52	−87.57	4.94
4	−78.61	−22.06	32.88	−108.27	−88.50	33.17
5	−86.40	−16.94	21.85	−94.48	−84.87	3.43
6	−89.80	−36.53	57.74	−111.28	−88.40	3.44
7	−91.80	−50.83	86.49	−129.45	−88.34	3.43
8	−109.42	−41.67	75.59	−124.28	−84.75	3.45
9	−92.66	−51.57	77.57	−106.80	−92.64	3.44
10	−96.30	−62.15	104.48	−129.26	−80.46	3.45

## Appendix F

**Table A6:** Measured pose of the marker relative to measurement frame for 10 sampled points in the experiment.

	$x_{MR}\;(\mathrm{mm})$	$y_{MR}\;(\mathrm{mm})$	$z_{MR}\;(\mathrm{mm})$	$\alpha_{MR}\;({}^{\circ})$	$\beta_{MR}\;({}^{\circ})$	$\gamma_{MR}\;({}^{\circ})$
1	299.41	−382.00	−2442.93	176.90	30.80	175.60
2	196.70	−396.14	−2394.80	175.60	6.14	172.62
3	160.22	−295.90	−2437.57	−175.82	6.90	175.46
4	442.53	−235.03	−2449.30	174.40	21.03	174.60
5	325.23	−255.80	−2393.16	−179.91	−1.13	172.97
6	231.40	−229.23	−2507.00	179.45	2.30	176.50
7	190.35	−234.70	−2662.45	175.70	4.42	176.69
8	−152.60	−305.32	−2594.70	179.66	21.90	172.80
9	159.96	−193.40	−2664.40	−171.67	4.71	−179.48
10	146.40	−246.80	−2815.70	−176.90	8.65	168.50

## Appendix G

**Table A7:** Joint angles of the robot before calibration for insertion points in the third row.

Holes	$\theta_{1}\;({}^{\circ})$	$\theta_{2}\;({}^{\circ})$	$\theta_{3}\;({}^{\circ})$	$\theta_{4}\;({}^{\circ})$	$\theta_{5}\;({}^{\circ})$	$\theta_{6}\;({}^{\circ})$
**t**_31_	−137.79	−49.57	103.69	−144.25	−89.99	−46.56
**t**_32_	−133.18	−52.97	111.16	−148.32	−90.00	−41.96
**t**_33_	−127.75	−55.74	117.37	−151.76	−90.01	−36.52
**t**_34_	−121.40	−57.87	122.23	−154.49	−90.03	−30.17
**t**_35_	−114.12	−59.33	125.63	−156.43	−90.04	−22.90
**t**_36_	−106.04	−60.10	127.45	−157.46	−90.06	−14.82

## Appendix H

**Table A8:** Joint angles of the robot after calibration for insertion points in the third row.

Holes	$\theta_{1}\;({}^{\circ})$	$\theta_{2}\;({}^{\circ})$	$\theta_{3}\;({}^{\circ})$	$\theta_{4}\;({}^{\circ})$	$\theta_{5}\;({}^{\circ})$	$\theta_{6}\;({}^{\circ})$
**t**_31_	−137.81	−49.58	103.67	−144.08	−89.84	−46.61
**t**_32_	−133.22	−52.99	111.16	−148.24	−89.78	−42.02
**t**_33_	−127.78	−55.75	117.38	−151.77	−89.73	−36.59
**t**_34_	−121.43	−57.87	122.26	−154.62	−89.71	−30.24
**t**_35_	−114.15	−59.32	125.67	−156.69	−89.71	−22.96
**t**_36_	−106.05	−60.06	127.49	−157.88	−89.75	−14.87

## References

[1] Lin Y., Zhao H., Ding H. (2017). Posture optimization methodology of 6r industrial robots for machining using performance evaluation indexes. Robot. Comput. Integr. Manuf..

[2] Judd R.P., Knasinski A.B. (1990). A technique to calibrate industrial robots with experimental verification. IEEE Trans. Robot. Autom..

[3] Nguyen H.N., Zhou J., Kang H.J. (2015). A calibration method for enhancing robot accuracy through integration of an extended Kalman filter algorithm and an artificial neural network. Neurocomputing.

[4] Nubiola A., Bonev I.A. (2013). Absolute calibration of an ABB IRB 1600 robot using a laser tracker. Robot. Comput.-Integr. Manuf..

[5] Wu Y., Klimchik A., Caro S., Furet B., Pashkevich A. (2015). Geometric calibration of industrial robots using enhanced partial pose measurements and design of experiments. Robot. Comput. Integr. Manuf..

[6] Gao G., Liu F., San H., Wu X., Wang W. (2018). Hybrid optimal kinematic parameter identification for an industrial robot based on bpnn-pso. Complexity.

[7] Jiang Z., Zhou W., Li H., Mo Y., Ni W., Huang Q. (2017). A new kind of accurate calibration method for robotic kinematic parameters based on the extended Kalman and particle filter algorithm. IEEE Trans. Ind. Electron..

[8] Borm J.H., Menq C.H. (1989). Experimental study of observability of parameter errors in robot calibration. International Conference on Robotics and Automation. IEEE.

[9] Driels M.R. (1993). Using Passive End-Point Motion Constraints to Calibrate Robot Manipulators. J. Dyn. Syst. Measur. Control.

[10] Lightcap C., Hamner S., Schmitz T., Banks S. (2008). Improved positioning accuracy of the PA10-6CE robot with geometric and flexibility Calibration. IEEE Trans. Robot..

[11] Nubiola A., Slamani M., Joubair A., Bonev I.A. (2014). Comparison of two calibration methods for a small industrial robot based on an optical CMM and a laser tracker. Robotica.

[12] He S., Ma L., Yan C., Lee C.H., Hu P. (2019). Multiple location constraints based industrial robot kinematic parameter calibration and accuracy assessment. Int. J. Adv. Manuf. Technol..

[13] Joubair A., Bonev I.A. (2015). Non-kinematic calibration of a six-axis serial robot using planar constraints. Precis. Eng..

[14] Joubair A., Bonev I.A. (2015). Kinematic calibration of a six-axis serial robot using distance and sphere constraints. Int. J. Adv. Manuf. Technol..

[15] Du G., Zhang P. (2013). Online robot calibration based on vision measurement. Robot. Comput. Integr. Manuf..

[16] Wang H., Lu X., Hu Z., Li Y. (2015). A vision-based fully-automatic calibration method for hand-eye serial robot. Ind. Robot Int. J..

[17] Meng Y., Zhuang H. (2007). Autonomous robot calibration using vision technology. Robot. Comput. Integr. Manuf..

[18] Zhang X., Song Y., Yang Y., Pan H. (2017). Stereo vision based autonomous robot calibration. Robot. Auton. Syst..

[19] Saund B., Devlieg R. (2013). High accuracy articulated robots with CNC control systems. SAE Int. J. Aerosp..

[20] Jiang Y., Huang X., Li S. (2016). An on-line compensation method of a metrology-integrated robot system for high-precision assembly. Ind. Robot Int. J..

[21] Shi X., Zhang F., Qu X., Liu B. (2016). An online real-time path compensation system for industrial robots based on laser tracker. Int. J. Adv. Robot. Syst..

[22] Yin S., Guo Y., Ren Y., Zhu J., Yang S., Ye S. (2014). Real-time thermal error compensation method for robotic visual inspection system. Int. J. Adv. Manuf. Technol..

[23] Guillo M., Dubourg L. (2016). Impact & improvement of tool deviation in friction stir welding: Weld quality & real-time compensation on an industrial robot. Robot. Comput. Integr. Manuf..

[24] Schneider U., Drust M., Ansaloni M., Lehmann C., Pellicciari M., Leali F., Gunnink J.W., Verl A. (2016). Improving robotic machining accuracy through experimental error investigation and modular compensation. Int. J. Adv. Manuf. Technol..

[25] Zeng Y., Tian W., Liao W. (2016). Positional error similarity analysis for error compensation of industrial robots. Robot. Comput. Integr. Manuf..

[26] Shu T., Gharaaty S., Xie W.F., Joubair A., Bonev I.A. (2018). Dynamic path tracking of industrial robots with high accuracy using photogrammetry sensor. IEEE/ASME Trans. Mechatron..

[27] Gharaaty S., Shu T., Joubair A., Xie W.F., Bonev I.A. (2018). Online pose correction of an industrial robot using an optical coordinate measure machine system. Int. J. Adv. Robot. Syst..

[28] Stückelmaier P., Grotjahn M., Fräger C. Iterative improvement of path accuracy of industrial robots using external measurements. Proceedings of the 2017 IEEE International Conference on Advanced Intelligent Mechatronics (AIM).

[29] Hsiao T., Huang P.H. (2017). Iterative learning control for trajectory tracking of robot manipulators. Int. J. Autom. Smart Technol..

